# Tuberculosis and pneumonia in HIV-infected children: an overview

**DOI:** 10.1186/s41479-016-0021-y

**Published:** 2016-11-24

**Authors:** Helena Rabie, Pierre Goussard

**Affiliations:** 1 Department of Pediatrics and Child Health, University of Stellenbosch and Tygerberg Academic Hospital, Cape Town, South Africa; 2 Childrens Infectious Diseases Clinical Research Unit (KidCRU), University of Stellenbosch, Cape Town, South Africa; 3 Division of Infectious Diseases, Department of Paediatrics and Child Health, Faculty of Medicine and Health Sciences, Stellenbosch University, PO Box 241, Cape Town, 8000 South Africa

**Keywords:** Human immunodeficiency virus, HIV, Children, Lower respiratory tract infection, Tuberculosis, Pneumonia

## Abstract

Pneumonia remains the most common cause of hospitalization and the most important cause of death in young children. In high human immunodeficiency virus (HIV)-burden settings, HIV-infected children carry a high burden of lower respiratory tract infection from common respiratory viruses, bacteria and *Mycobacterium tuberculosis*. In addition, *Pneumocystis jirovecii* and cytomegalovirus are important opportunistic pathogens. As the vertical transmission risk of HIV decreases and access to antiretroviral therapy increases, the epidemiology of these infections is changing, but HIV-infected infants and children still carry a disproportionate burden of these infections. There is also increasing recognition of the impact of *in utero* exposure to HIV on the general health of exposed but uninfected infants. The reasons for this increased risk are not limited to socioeconomic status or adverse environmental conditions—there is emerging evidence that these HIV-exposed but uninfected infants may have particular immune deficits that could increase their vulnerability to respiratory pathogens. We discuss the impact of tuberculosis and other lower respiratory tract infections on the health of HIV-infected infants and children.

## Background

To prevent vertical transmission of human immunodeficiency virus (HIV) from mother to child and to improve long-term outcomes for HIV-infected pregnant women, the current standard of care is to initiate lifelong suppressive antiretroviral therapy (ART) [1]. With this strategy, transmission should be less than 1% in women who either know their status, or are diagnosed early in pregnancy and are retained in care on an effective regimen [1, 2]. However, as vertical HIV transmission risk decreases there is increasing recognition of adverse health impacts suffered by HIV-exposed but uninfected (HEU) infants, including vulnerability to developing lower respiratory tract infections (LRTIs) and tuberculosis (TB) due to socioeconomic status and adverse environmental conditions such as maternal ill health and lack of breastfeeding [3]. In these infants there is also increasing recognition of immune dysfunction including immune activation, altered T-cell numbers and function, and possibly humoral immunity [3].

Despite major reductions in vertical HIV transmission among adequately treated mothers, large numbers of HIV-infected children continue to be born, especially in settings where HIV service delivery or maternal uptake of available services is sub-optimal. In 2013, it was estimated that globally there were 240,000 new pediatric HIV infections [4]. In the absence of antiretroviral therapy, perinatally infected infants and children have high morbidity and mortality, with 50% dying by the age of 2 years in low resource settings [5]. Despite the high early mortality without ART, many perinatally infected children have slowly progressive disease and some may only be diagnosed in late childhood or adolescence [6]. As established by clinical, epidemiological and postmortem studies, LRTIs are the major cause of morbidity and mortality in HIV-infected children. LRTIs in HIV-infected children are from common childhood respiratory pathogens (viral and bacterial), as well as opportunistic pathogens, such as *Pneumocystis jirovecii*, cytomegalovirus (CMV) and *Mycobacterium tuberculosis* [7–11].

## Tuberculosis

### Epidemiology

The overlap in the epidemiology of TB and HIV is well known, with its epicenter in sub-Saharan Africa. All of the countries with HIV prevalence of more than 10% in persons aged 15–49 years are in sub-Saharan Africa, and more than 90% of all HIV-infected children live in this region [3, 12, 13]. In addition, all of the countries with a TB incidence of more than 500 cases per 100,000 population are in sub-Saharan Africa, although large case numbers are also found in Asian countries with high population numbers [12]. In settings with a high burden of both TB and HIV, high rates of co-infection are reported and up to 56% of children treated for TB in sub-Saharan Africa are considered to be HIV-infected [8]. Studies of HIV cohorts confirm high rates of incident and prevalent TB. In Cape Town, South Africa, TB program data suggested a TB incidence of 1596 cases per 100,000 among HIV-infected infants prior to the implementation of universal ART [14], while clinical cohort data identified 53 cases per 100 patient-years in children not on ART [15]. A randomized-controlled trial to assess the effect of universal isoniazid preventive therapy (IPT) in HIV-infected children found a TB disease rate of 121 per 1000 patient-years in children who had early ART access [16].

Major differences in the number of children diagnosed in TB endemic settings with comparable adult TB incidence rates probably reflect varying ability to diagnose TB in young children [8]. Irrespective of the setting, it remains crucial to test all TB patients for HIV. This is highlighted by cohort reports from multiple settings; in the United Kingdom, 8 of 18 TB/HIV co-infected children presented with TB, which prompted their HIV diagnosis [17]. Studies of older children and adolescents in Zimbabwe found a high yield of new HIV diagnosis in adolescents presenting with TB [18]. Rates of TB are not only increased in HIV-infected infants, but evidence suggests that HEU infants are also at increased risk for TB. Data from South Africa suggest this risk may be 4-fold that of the general population [19], with rates of 41 cases per 1000 child-years reported in HEU infants [16].

Reasons for the high rates of TB in HIV-infected infants are multifactorial. HIV-infected and HEU infants and children are highly exposed to adult TB source cases. HIV-infected adults are 21–34 times more likely to develop TB in high burden settings, with TB accounting for up to a quarter of HIV-associated mortality [20]. Woman of child-bearing age are disproportionately affected [21], which implies a high risk of household TB exposure in vulnerable young HIV-infected and HEU children [22]. In a study of IPT in high TB burden settings, 10% of HIV-exposed infants had contact with potential source cases by the age of 14 weeks [23]. If TB in HIV-infected pregnant women is missed, the infants are not only at risk of TB, but have a 3-fold higher HIV acquisition rate [24]. Where there are no strategies to identify mothers with sputum smear-negative TB, there may be significant delays in diagnosis and extended exposure of young children to risk of infection [25, 26].

Alongside exposure and infection, young age is one of the most important risk factors for developing severe disease [27]. However, young age is poorly studied as a risk factor in HIV-infected children in resource-limited settings, but as many as 33% of infants starting ART at a median age of 8 months are already receiving anti-TB therapy [28]. Other risk factors are poor nutritional status (stunting, wasting and reduced mid-upper arm circumference), advanced immune suppression and anemia [8]. School attendance is also associated with disease in older children, reflecting poor ventilation in schools in low-resource, high-burden settings [22].

### Prevention

The most important tools we have to mitigate the HIV-associated risk of developing TB infection and disease are (i) access to ART for adults and children with HIV; and (ii) use of IPT. In the Children with HIV Early Antiretroviral Therapy (CHER) trial, conducted in Cape Town and Johannesburg, South Africa, TB occurred in 8.3% of children receiving early ART (initiated before12 weeks of age) and in 20% of children receiving delayed ART (commenced only after specific clinical and immunologic criteria were met) [29]. When ART is initiated in older children, reductions in presumed and confirmed incident TB of 32–70% are documented [8]. Nevertheless, HIV-infected children have more TB than their uninfected peers, including in low-burden settings [8, 30]. A transient increase in risk can also be expected in the first 3 months of therapy as part of immune reconstitution inflammatory syndrome (IRIS) [31, 32].

Though there is a decline in the number of children commencing ART only when they are severely immunocompromised, this situation is still common. In 2010 in Asia, Africa and South America, 63% of HIV-infected children in low-income countries, 66% in low–middle-income and 58% in upper–middle-income countries were already severely immunocompromised when commencing ART, compared with 19% of HIV-infected children in the United States [33]. Access to ART with broad penetration into the community will impact the epidemiology of TB, because there will be a decrease in adult cases of pulmonary disease. This is already clear from the decline in adult and pediatric TB cases in South Africa as the adult ART uptake increased. In Soweto, South Africa, ART access in TB in HIV-uninfected children declined from 18.7 to 11.0 per 100,000 [34].

In 2014, The Joint United Nations Programme on HIV/AIDS (UNAIDS) defined its ambitious 90–90–90 targets. The three targets to be achieved by 2020 are: 90% of HIV-infected persons must be diagnosed, 90% of those diagnosed should be on sustained therapy, and 90% of those on therapy should have an undetectable viral load [35]. If ART coverage expands to 80% of infected persons and all infected persons are eligible for therapy, the incidence of TB should decline by 28–37%. However, even if these targets are achieved, finding and treating TB cases remains important as it will prevent mortality and reduce TB transmission [36].

IPT is a well known prevention strategy in HIV-uninfected children and adults with known exposure to TB. In HIV-infected children there are no studies assessing post-exposure prevention; however, in a cohort of 494 children beginning ART when younger than 24 months of age in Cape Town, 127 courses of post-exposure IPT were provided, with only two children developing confirmed TB. Both had fully drug-susceptible isolates [28]. In a prospective study of prevention in older HIV-infected children, many with a history of prior TB infection, both all-cause mortality (16% in the placebo arm vs. 8% in the IPT arm) and incident cases of TB (9.9% vs. 3.8%) declined [37]. A subsequent reanalysis assessing the role of IPT and ART in these children found that the greatest reduction was in children receiving both ART and IPT [38]. In contrast, a pre-exposure prevention study enrolling very young infants found high rates of TB in HIV-infected and HEU infants, but no differences between those receiving IPT and placebo [16]. In this study, drug resistance was found to play an important role in IPT failure [39]. When present, children typically have primary rather than secondarily acquired multidrug-resistant (MDR) TB. HIV infection was independently associated with prevalent TB disease among child household contacts of adult MDR-TB cases and with failure of MDR preventative therapy [40]. In South African children with MDR-TB, HIV infection was reported in 54, 77, and 22% of cases from Johannesburg, Kwa-Zulu Natal, and Cape Town, respectively [41–43]. Reviewing the susceptibility/resistance patterns of adult contacts with TB is an essential component of managing children with HIV, irrespective of whether or not they have TB at the time. In children with MDR-TB, HIV is associated with a poorer outcome [44, 45].

The temporal associations between TB, influenza, and pneumococcal disease in children suggest a complex interaction between these infections. There is an increase in TB diagnosis approximately 3 months after the annual influenza season and a reduction in culture-confirmed TB in HIV-infected children following receipt of pneumococcal conjugate vaccines [46, 47]. The Antiretroviral Research for Watoto (ARROW) study followed 1,206 HIV-infected children for 5 years after ART and it included a randomized intervention to stop or continue co-trimoxazole after 96 weeks on ART. In this study, reduction in TB was associated not only with the duration of ART, but also with continued co-trimoxazole preventative therapy [48].

### Diagnosis

Pulmonary TB is the most common disease manifestation in HIV-infected children, with 85% diagnosed in an early series of hospitalized South African children [49]. However, both intra- and extra-thoracic disease may occur concurrently [49–52]. The principles of TB diagnosis include the following steps: 1) carefully assess TB contact history and/or seek proof of infection; 2) enquire about suggestive symptoms; and 3) seek evidence of active disease and bacteriologic confirmation. TB symptoms, though similar in HIV-infected and uninfected infants and children, overlap with symptoms of other infections and HIV itself. The chest radiograph may be very difficult to interpret, especially when there is underlying lung disease. TB may also be confused with acute pneumonia in HIV-infected children. It should also be remembered that HIV-infected children who have not yet been diagnosed with an underlying HIV infection may present to the health service with TB [6, 8, 15, 17, 18, 49, 50, 53].

### Treatment

The potential drug interactions between the TB therapy and ART are well known and, in general, TB therapy should not change. A dual treatment plan should consider the following questions:

1. What is the suspected or confirmed susceptibility pattern of *M. tuberculosis* and which drugs should be used?
2. Are alternative rifamycins to rifampicin available?
3. Which ART regimen is preferred AND can a suppressive regimen be given safely?
4. What are the potential drug interactions AND can adjustments be made if rifampicin is the only rifamycin available?

In the absence of ART, high mortality in HIV-infected children with TB was reported [49, 54] and continued with delayed ART initiation, especially when TB was the presenting diagnosis. Most deaths occurred within the first 2 months of delayed ART initiation and were not always directly TB-related [28].

Pharmacokinetic studies illustrate that both efavirenz and lopinavir-ritonavir 1:1 in their usual doses provide appropriate drug levels when co-administered with rifampicin [55, 56]. There are, however, ongoing concerns over nevirapine dosing and recommendations on dose adjustments have been suggested [57]. Table 1 summarizes important considerations when treating TB/HIV co-infection [58–61]. In co-treated children, efavirenz has not been associated with worse outcomes, but despite reassuring data on the pharmacokinetic outcomes of lopinavir-ritonavir 1:1 concurrent TB therapy may be a risk factor for failure in children on lopinavir-ritonavir treatment [62–64]. The World Health Organization (WHO) also recommends a non-suppressive regimen of zidovidine, lamivudine and abacavir in children needing rifampicin as a short-term measure to avoid drug interactions [65]. This is based on the ARROW study. In ARROW, three nucleoside/nucleotide reverse transcriptase inhibitors (NRTIs) were studied as an intensive induction and maintenance strategy when combined with non-nucleoside reverse transcriptase inhibitors (NNRTIs). Fifty-nine of the 1143 children required a drug alteration for TB that included stopping or replacing the NNRTI nevirapine. In contrast, children taking efavirenz did not require a drug switch. Triple NRTI for HIV suppression was effective at 36 weeks, but not at 144 weeks. The advantage of this latter strategy is that, although viral suppression is inferior, the risk of progressive NNRTI and protease inhibitor (PI) ART resistance was avoided and drug–drug interactions and logistic complications were minimized [66]. There is no current guidance on using integrase inhibitors and other PIs such as darunavir in children, and no data on newer anti-TB agents such as bedaquiline and delamanid in HIV-infected children. Nevertheless, ART should be initiated 2–8 weeks after starting TB therapy so as to reduce the risk of IRIS occurring [61]. For example, in TB meningitis cases deterioration due to paradoxical IRIS can be devastating and ART should be delayed at least 4 weeks [67, 68]. In Table 2 we highlight key messages to consider in TB and HIV co-infected children. TB may be an important challenge and opportunity in achieving the UNAID 90–90–90 targets [69].

**Table 1:** Summary of the first and second line anti-tuberculosis medications with recommended doses and drug-drug interactions* [60–63]

Drug	Dose Recommended	Drug-drug interactions with antiretrovirals
Group 1		
Isoniazid	7–15 mg/kg once daily	None
Rifampicin	10–20 mg/kg once daily	Coadministration reduces concentrations of^a^ NNRTIs, ^b^PIs, integrase inhibitors
Pyrazinamide	30–40 mg/kg once daily	None
Ethambutol	15–25 mg/kg once daily	None
Rifabutin	10–20 mg/kg/day (Max Dose 300 mg/day)	Boosted PI: increase rifabutin levels and rifabutin dose reduction is needed NNRTI: Efavirence reduces the concentration of rifabutin, increasing the rifabutin dose is recommended in adults Nevirapine dose adjustment is not needed for rifabutin
Group 2		
Kanamycin	15–30 mg/kg once daily, max 1 g	
Amikacin	15–22.5 mg/kg once daily, max 1 g	
Capreomycin	15–30 mg/kg once daily, max 1 g	
Streptomycin	20–40 mg/kg once daily, max 1 g	
Group 3		
Ofloxacin	15–20 mg/kg once daily, max 800 mg	
Levofloxacin	(15–20 mg/kg once daily)†, 7.5–10 mg/kg once daily, max 750 mg	
Moxifloxacin	7.5–10 mg/kg once daily, max 400 mg	Moxifloxacin concentration could be reduced by ritonavir, though limited data; buffered didanosine may reduce oral absorption of all fluoroquinolones
Group 4		
Ethionamide/Prothionamide	15–20 mg/kg once daily, max 1 g	Possible, unknown
Cycloserine/Terizidone	10–20 mg/kg once or twice daily, max 1 g	Unlikely, unknown
Para-aminosalicylic acid (PAS)	150 mg/kg granules daily in 2–3 divided doses, max 12 g	Co-administration with efavirenz may reduc PAS AUC by 50%
Group 5		
Linezolid	(10 mg/kg twice daily, once daily for >10 years of age)^c^	Unlikely
Clofazimine	(3–5 mg/kg once daily)^c^	Unknown; may be a weak CYP3A4 inhibitor
Amoxicillin-clavulanic acid, Meropenem-clavulanic acid, and Imipenem/cilastin	As for bacterial infections	Unlikely
Thiacetazone	5–8 mg/kg once daily	Contraindicated in HIV-infected individuals
High-dose isoniazid	15–20 mg/kg once daily	None
Clarithromycin	7.5–15 mg/kg twice daily	Clarithomycin levels increase with boosted atazanavir and lopinavir with increased risk of toxisity. Clarithomycin levels are decreased by efavirence, nevirapine and etravirine Azithromycinn is prefered
Azithromycin	10 mg/kg once daily	Prefered macrolide but limited activity and caution required

† Indicates bracketed recommended by some experts, but differs from formal WHO guideline

^a^ *NNRTI* Non-nucleoside reverse transcriptase inhibitor, ^b^ *PI* Protease inhibitor

^c^No formal paediatric dose recommended in WHO guidelines, so presented dose based on experience and expert opinion

**Table 2:** Summary of key messages related to TB/HIV co-infection in children

HIV-infected and exposed uninfected (HEU) children are at high risk of developing TB	High likelihood of household TB exposure early in life; disproportionate TB/HIV burden in young mothers
	Advanced disease, low CD4 count and malnutrition are additional risk factors
	All children with HIV must be regularly screened for TB exposure and disease
	BCG vaccination is contra-indicated in children known to be HIV-infected
Strategies to prevent TB	High community penetration of adult ART will reduce TB transmission in communities
	Universal early ART initiation in all HIV-infected
	Appropriate use of INH preventative therapy
	Active TB Case finding
	Infection prevention and control strategies
	History of contact
Early diagnosis of TB	Symptoms can overlap with other opportunistic infections
	Pulmonary and extrapulmonary disease possible
	Pulmonary TB can present like acute pneumonia
	A positive TST (≥5 mm) or IGRA confirms TB infection but not disease
	CXR is important; HIV-infected children more likely to have alveolar opacification and lung cavities
	Collect specimens for culture and Xpert MTB/RIF® as appropriate
Treating TB/HIV co-infection	Use appropriate/standard TB treatment regimen
	Adapt the ART regimen and dosing
	Support adherence
	Monitor for efficacy and side effects

*HIV* human immunodeficiency virus, *TB* tuberculosis, *ART* antiretroviral treatment, *INH* isoniazid, *TST* tuberculin skin test, *IGRA* interferon gamma release assay, *CXR* chest radiograph

### Pneumonia

Pneumonia remains the leading cause of pediatric mortality and morbidity. Globally, there are approximately 12–14 million hospitalizations and 0.68–0.92 million deaths annually from pneumonia in children [70–72]. HIV-infected children are at increased risk of pneumonia that requires hospital admission and death from this infection. In a meta-analysis and modelling study, Theodoratou and colleagues [7] calculated that in 2010 there were 0.6–3.3 million pneumonia episodes and 47,400–153,000 pneumonia deaths in HIV-infected children living in low-resource settings. Of these cases, 1.3 million (90%) pneumonia episodes and 93% of the deaths occurred in children younger than 5 years of age from the Africa region. HIV-infected children had a 6.5 (95% CI 5.9–7.2) increase in odds of pneumonia that required hospitalization and they had an increased risk of death (odds ratio 5.9, 95% CI 2.7–12.7) [7]. The authors showed that there is also a wide variation between countries; for example, in Zimbabwe 55% of all pneumonia deaths are attributed to HIV, compared to 1% in India [7].

Even with early access to ART, pneumonia remains common among HIV-infected infants and children. In the CHER study, pneumonia occurred at a rate of 12.2 infections/100 person-years in children accessing ART before 12 weeks of age and 26.5 infections/100 person-years in children accessing ART only after laboratory evidence of immune suppression and/or clinical features of HIV infection became apparent [29]. Poor general health and malnutrition, immune depletion, chronic lung disease, increasing household exposure and vaccine responsiveness may all contribute to the risk of LRTI. A wide range of pathogens causing LRTIs and pneumonia in children with HIV is reported [73]. The importance of polymicrobial infections—including combinations of bacterial, viral, *P. jirovecii* and mycobacterial infections—is well recognized. Mortality is increased significantly with increasing numbers of pathogens. Children with polymicrobial pneumonia have a 10-fold greater risk of dying than children in whom only a single organism is identified [74].

### Acute bacterial pneumonia

HIV-infected children have a higher risk of developing bacterial pneumonia than uninfected children. This remains a significant problem, even after the introduction of ART. HIV-infected children with pneumonia are more likely to have severe disease (including bacteremia), more complications, higher rates of treatment failure and death, and possibly more antimicrobial resistance than HIV-uninfected children [74]. The most common cause of bacterial pneumonia remains *Streptococcus pneumoniae*, but *Staphylococcus aureus* is increasingly important. Amongst Gram-negative bacteria associated with pneumonia in HIV-infected children, *Klebsiella* species, *Haemophilus influenzae*, *Escherichia coli, Pseudomonas aeruginosa* and non-typhoidal *Salmonella* species are also important [73].

HIV does not affect the radiographic appearance of bacterial pneumonia. The two classic patterns of either diffuse patchy air-space disease of bronchopneumonia, or confluent air-space disease with air bronchograms of lobar or segmental pneumonia can be found [75]. Necrotizing pneumonia, a severe complication of community-acquired pneumonia in non-HIV infected children, is characterized by liquefaction and cavitation of lung tissue and is being seen increasingly [76, 77]. The most common cause is *S. pneumoniae,* and although in the authors’ experience it also occurs in HIV-infected children, this has not been reported to date in the literature. The chest radiographs in necrotizing pneumonia frequently demonstrate both pleural and parenchymal involvement. The so-called ‘atypical pneumonias’ can present with a variable radiographic appearance, which includes reticulonodular infiltration, patchy or confluent air-space disease or a combination of all three patterns [78]. It is, therefore, very important to correlate the radiographic appearance with the clinical setting [78].

Without appropriate vaccination or ART, HIV-infected children experience a 40-fold increase in the risk of pneumococcal bacteremia, pneumonia and meningitis compared to vaccinated HIV-uninfected children, and they also often experience multiple episodes of invasive disease [79]. In South Africa, ART decreased the pneumonia burden by half with an even higher (65.2%) decline in mortality from invasive pneumococcal disease [80]. Vaccinating with the pneumococcal conjugate vaccine causes a significant reduction in vaccine-type serotype colonization and disease [79–81]. The reported increases in empyema with non-vaccine serotypes are mostly from areas with very low incidence of HIV and the real incidence of empyema in HIV-positive children is not known [82].

### Viral pneumonia

Viral infections play a major role in LRTIs and viruses are commonly identified in HIV-infected children hospitalized with these infections. Viral LRTIs may also play an important role in the pathogenesis of bacterial pneumonia and in the epidemiology of TB. In a large study from South Africa at least one virus was identified in 68% of HIV-infected children younger than 5 years of age and in 58% of those aged 5–14 years presenting with symptoms of a LRTI. More than one respiratory virus was found in 28 and 19% of children within these age groups, respectively. In children younger than 5 years the most common viruses detected were human rhinovirus (36%), adenovirus (32%), respiratory syncytial virus (RSV) (13%), enterovirus (8%), parainfluenza (9%) and influenza viruses (7%) [83, 84]. In addition, human bocavirus, human polyomaviruses and human coronaviruses are increasingly found in children with LRTIs, including those with HIV [85]; however, it is difficult to attribute causality to these viruses [85].

RSV is a very important pathogen in infants and young children and a common cause of LRTIs that require healthcare visits and hospitalization. HIV is an important risk factor for severe RSV infections, especially in high-burden settings [86]. HIV-infected children are older and may develop RSV outside of the seasonal peaks. Compared with HEU children, there is an increased incidence of hospitalization (3–5-fold) in children with HIV, and they tend to stay in hospital longer and have a higher risk of death. In a South African study of RSV-associated LRTI infection, 49 of the 1157 children with confirmed RSV were HIV-infected. In this study, ART data was only available for 12 cases, 6 of whom received ART. Four of the 9 deaths in this study occurred in HIV-infected children; however, their ART data are unknown [87].

Influenza is an important cause of pneumonia hospitalization and death. ART naïve HIV-infected children are up to 8 times more likely to be hospitalized and have a higher case fatality rate (8% in HIV-infected vs. 2% in uninfected children) [88, 89]. In a prospective South African study, pandemic and seasonal H1N1 influenza had a similar clinical course in HIV-infected children [90]. Giannattasio et al. reported on 11 HIV-infected and 30 otherwise healthy children hospitalized for H1N1 influenza. Leukopenia was recorded in 64% of HIV-infected and in 20% of healthy children (*p* = 0.01). Interstitial pneumonia was more frequent in HIV-positive children and consolidation was more frequent in HIV-negative children. Overall, 91% of HIV-positive children received oseltamivir. The H1N1 influenza attack rate was very high (20%) in HIV-infected children, but it consistently ran a mild course [91]. Annual vaccination with the seasonal influenza vaccine is recommended, but efficacy may be influenced by decreased vaccine immunogenicity reported in some studies [92].

CMV is a common co-infection in HIV-infected children and is frequently isolated from urine and respiratory secretions [93, 94]. Congenital and post-partum acquisition of CMV is common in HIV-exposed and infected infants and CMV infection may be associated with breastfeeding acquisition of HIV [95, 96]. The role of co-infection with CMV in children, especially those suspected of having *P. jiroveci* pneumonia (PJP), is poorly understood, but CMV itself can cause fatal pneumonia [94, 97]. Postmortem studies from Africa reported that in the 0–5 months age group, CMV (42.1%) was the second most common cause of fatal pneumonia after *P. jirovecci* (51.3%) [9]. The isolation of CMV by culture or polymerase chain reaction (PCR) from various sites or specimens does not prove that the child has CMV pneumonia. The chest radiographic appearance of PJP and CMV pneumonia can be very similar (diffuse alveolar disease). In children being ventilated for PJP and not responding to treatment for PJP, CMV was histologically proven in 72% of cases and associated with a poor outcome [97, 98]. CMV viremia is common in all infants with pneumonia, and a viral logarithmic load (to the base 10) of 4.6 or more was predictive of pneumonia in a study of from Cape Town [99]. In HIV-infected infants with severe pneumonia from suspected PJP, we would suggest that empiric treatment for CMV infection with either ganciclovir or valgancyclovir be used wherever possible, until such time that CMV infection can be excluded.

Despite access to effective vaccination, measles remains an important pathogen in children [100]. In settings with a high HIV burden, a significant number of children hospitalized with measles are also infected with HIV—such as Zambia, where up to 17% of hospitalized children with measles had HIV infection [101]. HIV-infected children with measles are significantly younger than uninfected children and often have not yet received the first vaccination of their primary measles containing vaccine schedule, even if that is scheduled at 9 months of age [102, 103]. Consequently, the WHO recommends first dose of a measles containing vaccine be administered at the age of 6 months in HIV-infected children [104]. Measles is generally thought to be more severe in HIV-infected children—both the duration of illness prior to hospitalization and the duration of hospitalization is longer [102, 103]. In low-resource settings, the case fatality rate is 2–7-fold higher in children with HIV [103, 104]. The effect of ART in preventing measles or modifying its severity is not fully understood, but initiation of ART at the time of admission for measles did not prevent readmission during a recent large measles outbreak in Cape Town [102].

### *Pneumocystis jiroveci* pneumonia

*P. jirovecii* is a common and important pathogen in HIV-infected children and causes a severe, rapidly progressive pneumonia with a high mortality rate despite access to antibiotic therapy [93, 94]. This pathogen is also increasingly reported in severe pneumonia in young HEU infants [105, 106]. The chest radiograph findings show increased lung volumes (78%) and diffuse parenchymal opacification (64%) that lead ultimately to diffuse alveolar opacification (92%). This usually happens within 72 h of presentation [107]. Clinically, lack of fever, high respiratory rate, low oxygen saturation and absence of co-trimoxazole preventative therapy is suggestive of PJP. Real-time PCR is better than immunofluorescence at making a microbiologic diagnosis [108]. When PJP is suspected, high-dose co-trimoxazole and steroids should be added to the management of severe LRTI.

The most important strategy to prevent PJP is by using co-trimoxazole prophylactic therapy from 6 weeks of age [109]. Stopping co-trimoxazole after CD4 recovery was studied in African children, and its continuation may have additional benefits beyond PJP prophylaxis, including TB prevention [48].

## Conclusions

Acute respiratory compromise caused by *M. tuberculosis* and other lung pathogens is a major cause of disease and death in HIV-infected children. Furthermore, many also develop chronic lung disease with bronchiectasis due to recurrent lung infections [110, 111]. In children with delayed ART initiation, nearly a third (28%) develop chronic lung disease with significantly compromised lung function [112]. This highlights the need for early ART initiation and universal ART access for all HIV-infected children to reduce the high burden of lung disease. Finally, vaccination, appropriate use of preventive therapy and early effective treatment of lung infections, including TB, are also essential to ensure optimal lung health in HIV-infected children.

## Abbreviations

ARROW: The Antiretroviral Research for Watoto
ART: Antiretroviral therapy
CHER: Children with HIV early antiretroviral
CMV: Cytomegalovirus
CXR: Chest radiograph
HEU: HIV-exposed but uninfected infants
HIV: Human immunodeficiency virus
IGRA: Interferon gamma release assay
IPT: Isoniazid preventive therapy
IRIS: Immune reconstitution inflammatory syndrome
LRTI: Lower respiratory tract infections
MDR: Multidrug-resistant
NNRTI: Non-nucleoside reverse transcriptase inhibitors
NRTI: Nucleoside/nucleotide reverse transcriptase inhibitors
PCR: Polymerase chain reaction
PI: Protease inhibitor
PJP: *P. jiroveci* pneumonia
RSV: Respiratory syncytial virus
TB: Tuberculosis
TST: Tuberculin skin test
UNAIDS: The Joint United Nations Programme on HIV/AIDS
WHO: World Health Organization
